# Secondary organizing pneumonia following viral pneumonia caused by severe influenza B: a case report and literature reviews

**DOI:** 10.1186/s12879-017-2677-1

**Published:** 2017-08-15

**Authors:** Nobuhiro Asai, Toyoharu Yokoi, Naoya Nishiyama, Yusuke Koizumi, Daisuke Sakanashi, Hideo Kato, Mao Hagihara, Hiroyuki Suematsu, Yuka Yamagishi, Hiroshige Mikamo

**Affiliations:** 1 Department of Clinical Infectious Diseases, Aichi Medical University School of Medicine, 〒480-1195 1-1 Yazakokarimata, Nagakute, Aichi Japan; 2 Department of Pathology, Nagoya Ekisaikai Hospital, Nagoya, Aichi Japan

**Keywords:** Influenza pneumonia, Type B influenza virus, Organizing pneumonia

## Abstract

**Background:**

Some reported that organizing pneumonia (OP) may occur after influenza A infections including swine-origin influenza A (H1N1). However, OP associated with influenza B infection has never been reported. We report the first case of secondary OP associated with viral pneumonia caused by influenza B.

**Case presentation:**

A 23-year old woman was diagnosed as viral pneumonia caused by type B influenza. Despite of antiviral therapy, abnormal chest shadows were not improved. Bronchoscopy and transbronchial lung biopsy showed organizing pneumonia due to viral pneumonia caused by influenza B. Corticosteroid therapy was started at 30 mg daily (0.5 mg/kg), and the dose was reduced to 25, 20, 15 or 10 mg per day every month with symptomatic and radiological resolution. Even after corticosteroid therapy was discontinued, we did not confirm disease recurrence.

**Conclusions:**

Physicians should be aware of the possibility for SOP and severe viral pneumonia even in case of type B as well as type A influenza infections.

## Background

Organizing pneumonia (OP) is a pulmonary inflammatory condition that occurs in certain clinical settings [1, 2]. In the appropriate clinical-radiologic context, an OP diagnosis can be confirmed with bronchoalveolar lavage fluid (BALF) and transbronchial lung biopsy (TBLB). Some reported that OP may occur after influenza A infections including swine-origin influenza A (H1N1) [3–5]. However, OP associated with influenza B infection has never been reported. We report the first case of secondary OP associated with viral pneumonia caused by influenza B.

## Case presentation

**Fig. 1:**
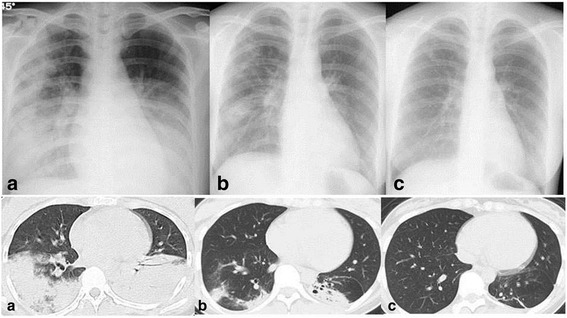
Chest X-ray showed bilateral infliltrates on admission (**a** upper). After starting PSL therapy on day 25, infiltrates were improved (**b** upper). Abnormal shadows on Chest X-ray disappeared 6 months after starting corticosteroid therapy (**c** upper). Chest CT showed consolidations on both lungs on admission (**a** lower) and the shadows were improved on day 25 after starting corticosteroid therapy (**b** lower). Six months after starting corticosteroid therapy, the consolidations disappeared (**c** lower)

**Fig. 2:**
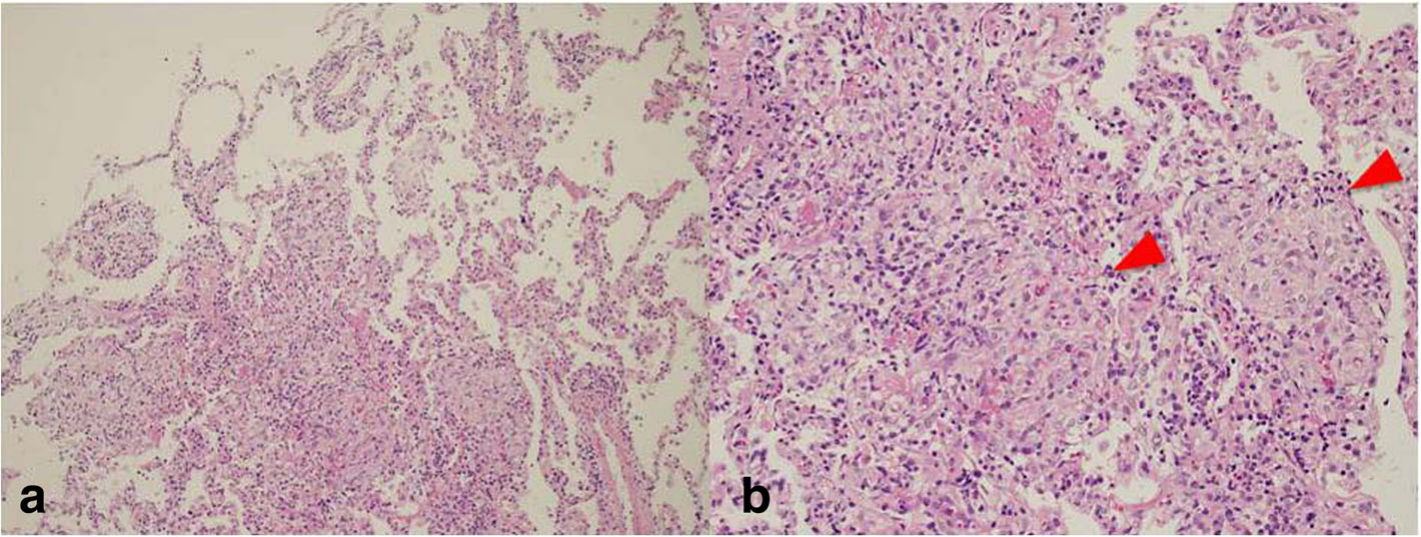
TBLB specimen from the right middle lobe showed intraalveolar granulation tissue with myofibroblasts consistent with organizing pneumonia (Hematoxin-Eosin (HE) × 100) (**a**). Masson body (red arrow) was seen on the TBLB specimen (HE ×400) (**b**)

A 23-year old woman without relevant medical history visited our outpatient clinic complaining fever, sorethorat and cough lasting for 3 days. She was diagnosed as influenza B infection by nasophayngeal antigen test (Influenza B strain was not confirmed because reverse transcription-polymerase chain reaction was not performed.). She was not obese (body mass index 22.3 kg/m^2^) and had not received any influenza vaccination in the past. She had no smoking history. She had an allergy history for macrolides antibiotics. Although she had received an antiviral therapy of oseltamivir 150 mg per day, flu symptoms such as fever and cough persisted and she once again came to the clinic on day 4 after starting the therapy. Also, she had an acute respiratory failure (SpO_2_ 93% on 5 L/min O_2_ mask) with elevation of inflammatory reactions (WBC 18,900 /μL, CRP 11.69 mg/dL). Chest radiography and chest computed tomography (CT) revealed peripheral subpleural opacities (Fig.1) and consolidations. Broad spectrum antimicrobial agent, meropenem, was empirically administered but additional antiviral therapy was not started. A rapid influenza diagnostic test showed that influenza A and B were negative and positive, respectively. Urinary streptococcal antigen and legionella antigen were both negative. No virulent pathogens were detected in the sputum. Therefore, she was diagnosed as having influenza B virus pneumonia and was referred to us. While her respiratory condition was improved, inflammatory reaction and abnormal chest radiographic shadows recurred. Transbronchial lung biopsy (TBLB) revealed lymphocytic alveolitis and organizing pneumonia (Fig 2). No pathogens were found in the BALF. She was diagnosed as secondary OP associated with influenza B pneumonia. Corticosteroid therapy was started and inflammatory reaction and chest radiography were gradually improved. The initial dose of corticosteroid was 30 mg (0.5 mg/kg) daily and tapered with improvement of inflammatory reaction and chest radiography. She was discharged on day 30. The dose was reduced to 25, 20, 15 and 10 mg per day every month with symptomatic and radiological resolution. After corticosteroid therapy was discontinued, disease recurrence was not observed.

## Discussion

In general, influenza B complicating bacterial infection cause a mild disease, but severe pneumonia cases due to influenza B and *Streptococcal* co-infection have been reported [6–8].

Several mechanisms of such severe co-infection were reported in animal models. The influenza virus could damage respiratory tract epithelium and facilitate bacterial infection. The viruses also alter and/or impair ciliary function of the epithelium. It can change host immunity and inflammatory responses by impairing bacterial clearance or via the inflammatory cascade [9–11]. To the best of our knowledge, this is the first case of SOP due to influenza B virus pneumonia and the second case of severe respiratory failure due to influenza B virus infection not co-infection, following the case reported by Kato [12]. Gutierrez-Pizarraya, et al. reported that the proportion of patients with pneumonia and the rate of admission to the intensive care unit did not differ between cases of influenza A (H1N1) pdm09 and influenza B infection. Notably, the mortality rates were almost similar between patients with influenza A (16.3%) and influenza B (10%) infection [13]. Paddock, et al. documented the pathological findings of the lung autopsy cases of patients who died of influenza B virus infection and demonstrated that diffuse alveolar damage pattern were found in 17.8% of the cases [14]. These suggest that influenza B virus infection could contribute to severe respiratory failure, resulting in poor outcome that is similar to influenza A virus infection.

The standard treatment of influenza virus pneumonia is not established yet. Tanaka reported that the median survival time of post-influenza pneumococcal pneumonia in mice was longer by double dose of peramivir than by single dose of peramivir or single dose of oseltamivir group. Also, the production of inflammatory cytokines/chemokines was also significantly suppressed by double dose of peramivir compared with other two groups. These suggests that double dose of peramivir could contribute to reducing a cytokine storm by influenza virus, resulting in favorable outcome**s** for influenza virus pneumonia and post-influenza pneumonia patients [15]. In contrast, some demonstrated that double dose of neuraminidase inhibitors are not useful in the treatment for flu compared with the standard dose of oseltamivir [16]. In the study, they concluded that the duration of antiviral therapy was associated with the outcome. Other studies also indicate that early antiviral therapy in patients with severe influenza is associated with both clinical benefits and more rapid viral clearance from the upper respiratory tract samples [17, 18].

Some previously reported cases of SOP caused by influenza virus (Table 1) [3–5, 7, 8]. It is well known that H1N1 and avian flu could result in a cytokine storm. Thus, it is reasonable that SOP could occur in patients infected with these viruses. In terms of radiological findings of influenza A pneumonia, peripheral ground-grass opacities and consolidations has been commonly reported as in our case. Some documented a peripheral distribution of lung opacities in patients with influenza A infection mimicking OP [19–21]. SOP could be seen 2 to 3 weeks after the onset of initial influenza symptoms, whereas influenza viral pneumonia could generally occur in day 4 to 5 after the onset of illness [22, 23]. SOP should be considered if influenza patients have fever, and a dry cough after initial influenza like illness improved, and TBLB should be performed since proper OP treatment requires corticosteroid therapy. All SOP cases following severe influenza virus pneumonia were cured by corticosteroid therapy, except for the case of acute fibrosis organizing pneumonia (AFOP) associated with influenza A/H1N1 pneumonia after lung transplantation [8] as shown in Table 1. These favorable outcomes of SOP could correlate with the clinicopathological features of OP. Such features of OP might be different from that of a fatal interstitial lung disease such as diffuse alveolar damage. Ebina and colleagues reported the disappearance of subpleural and interlobular lymphatics in idiopathic pulmonary fibrosis (IPF) lungs along with a poor lymphogenesis and a significant decrease of alveolar lymphangiogenesis in comparison with cellular non-specific interstitial pneumonia and OP. These changes may exert a detrimental effect on IPF lungs by impairing alveolar clearance [24].

**Table 1:** Cases of secondary organizing pneumonia due to influenza virus infection

Author (Year)	Sex	Age	Type of influenza	Initial treatment	Outcome
Cornejo R [3] (2010)	Female	52	A	Steroidpulse therapy	Cure
Cornejo R [3] (2010)	Male	36	A	Steroidpulse therapy	Cure
Gómez-Gómez A [4] (2011)	Female	44	A	Corticosteoroid 0.5 mg/kg	Cure
Gómez-Gómez A [4] (2011)	Male	60	A	Corticosteoroid 0.5 mg/kg	Cure
Torrego A [5] (2010)	Female	55	A	Corticosteoroid 0.75 mg/kg	Cure
Kwok WC [7] (2016)	Female	45	B	Corticosteroid 30 mg daily	Cure^a^
Otto C [8] (2013)	Female	66	B	High dose corticosteroid	Death^b^
Asai N (2016)	Female	22	B	Corticosteroid 0.5 mg/kg	Cure

All cases were diagnosed as organizing pneumonia by transbronchial lung biopsy

^a^ This is a case of secondary organizing pneumonia associated with influenza B and *Streptococcal* co-infection

^b^ This is a case of acute fibrinous and organizing pneumonia associated with influenza A/H1N1 pneumonia after lung transplantation

AFOP is a distinct reaction pattern in acute respiratory failure with high mortality rate of up to 90%. Current literature reported that cause of AFOP are idiopathic, or collagen vascular diseases, or bacterial infection, drug exposure (e.g. amiodarone, abacavir or statins) [8, 25]. Physicians should know that influenza infection could induce AFOP, resulting in a poor outcome.

## Conclusions

Physicians should be aware of the possibility of severe respiratory failure due to influenza B virus infection and secondary OP following influenza B infection, even though once influenza infection was cured.

## Abbreviations

BALF: Bronchoalveolar lavage fluid
CT: Computed tomography
IPF: Idiopathic pulmonary fibrosis
OP: Organizing pneumonia
TBLB: Transbronchial lung biopsy
